# Orbital Onslaught: An Atypical Presentation of Mucormycosis in a Diabetic and Hypertensive Patient

**DOI:** 10.7759/cureus.49658

**Published:** 2023-11-29

**Authors:** Vyshnavidevi Sunkara, Muhammad Abubakar, Syeda Aelia Husain Hamdani, Han Grezenko, Thowaiba E Ali

**Affiliations:** 1 Internal Medicine, Katuri Medical College, Guntur, IND; 2 Internal Medicine, Wah Medical College, Wah Cantt, PAK; 3 Medicine, Ghurki Trust and Teaching Hospital, Lahore, PAK; 4 Translational Neuroscience, Barrow Neurological Institute, Phoenix, USA; 5 Healthcare Administration, University of Tennessee at Chattanooga, Chattanooga, USA

**Keywords:** disease management, atypical presentation, orbital cellulitis, immunocompromised, surgical debridement, antifungal therapy, angioinvasive fungal disease, diabetic complications, orbital infection, mucormycosis

## Abstract

Mucormycosis, primarily known to affect the sinuses and brain, severely threatens immunocompromised individuals. Its occurrence in the orbital region is rare and potentially devastating.

We report a unique case of a 50-year-old male with longstanding diabetes and hypertension who exhibited isolated orbital mucormycosis confined to the right eye. The patient presented with fever and acute vision loss without classic sinusitis symptoms; earlier mismanagement led to an aggressive relapse. An orbital computed tomography (CT) scan revealed inflammatory changes indicative of an early-stage invasive fungal infection. Comprehensive management involving surgical debridement and antifungal therapy successfully halted intracranial spread and further complications.

This case underscores the necessity for high clinical vigilance in diagnosing atypical presentations of mucormycosis in susceptible populations, advocating for a rapid, multidisciplinary approach to ensure optimal outcomes. It also adds to the existing literature on the myriad manifestations of this formidable fungal infection.

## Introduction

Mucormycosis, commonly known as "black fungus," is caused by mucormycetes molds and poses a significant risk, especially to immunocompromised individuals with diabetes or a history of organ transplants. The infection's aggressive nature necessitates awareness and prompt action in high-risk groups, which can quickly become life-threatening [1].

Despite its rarity, the severity of mucormycosis cannot be overlooked. With a high mortality rate that can range from approximately 40% to 80%, depending on the site of infection and the patient's underlying conditions, its rapid progression underscores the importance of early detection and immediate antifungal treatment, particularly in susceptible individuals. Timely intervention is crucial to prevent serious outcomes, including but not limited to widespread tissue necrosis, invasion of the infection into the cranial structures leading to rhinocerebral mucormycosis, loss of vision, and dissemination to other parts of the body, which can result in multi-organ failure [2,3].

The prevalence of mucormycosis varies globally, ranging from 0.005 to 1.7 cases per million. This variability, influenced by geographic and environmental factors and population-specific risk factors, highlights the need for effective region-specific public health strategies to manage and prevent infection [3]. The primary objective of this case report is to heighten awareness surrounding this infrequent fungal infection. By detailing its salient clinical features, diagnostic hurdles, effective management strategies, and patient outcomes, we aim to underscore the necessity for prompt recognition, accurate diagnosis, and timely treatment of this potentially vision- and life-compromising condition.

## Case presentation

A 50-year-old patient with a history of diabetes and hypertension arrived at the hospital, presenting with a six-day history of fever and five-day right eye swelling. His medical records indicated poorly controlled diabetes, with an HbA1c level of 8.5%, and hypertension, with recent readings showing a systolic blood pressure of 150 mmHg and a diastolic pressure of 95 mmHg. The fever was sudden, high-grade, and intermittent, without rigors or chills. Subsequently, his right eye developed swelling, revealing a black mass accompanied by blurred vision but no discharge. A month earlier, he was diagnosed with mucormycosis at another hospital based on a clinical evaluation. Despite receiving a week's treatment, his symptoms quickly recurred, leading to his visit to our facility.

Upon presentation, the most striking feature was the marked swelling of the patient's right eye. The swelling had formed a black eschar, a dead piece of tissue that typically hardened and darkened. The black eschar, indicative of significant tissue necrosis, is a hallmark of advanced mucormycosis. This darkening is primarily due to ischemia-induced tissue death, where the lack of blood supply leads to necrosis. The absence of any discharge was notable, though the blurring of vision was a concerning symptom that could indicate potential damage to the ocular structures or an indication of the fungal invasion's progress. Furthermore, aside from the evident ocular manifestations, the general physical examination was unremarkable. There were no signs of systemic involvement or other complications, making it crucial to decipher the nature and extent of the local infection.

In light of the presentation and physical findings, a comprehensive set of investigations was initiated to better understand the underlying etiology and any potential complications. A complete blood workup has been provided in Table 1.

**Table 1:** Complete blood workup of the patient. INR: international normalized ratio, APTT: activated partial thromboplastin time, WBC count: white blood cell count, RBC: red blood cells, HCT: hematocrit, MCV: mean corpuscular volume, MCH: mean corpuscular hemoglobin, MCHC: mean corpuscular hemoglobin concentration, ALT: alanine transaminase, AST: aspartate aminotransferase, ALP: alkaline phosphatase.

Coagulation Profile	Patient value	Reference range
Prothrombin Time-Control	12	10-14 seconds
Prothrombin Time-Patient	11	Up to 13 seconds
INR	1.2	0.9-1.3
Control Time	30	25-35 seconds
APTT	28	Up to 31 seconds
Hemogram		
WBC count	12	4-11 x10^9^/L
Total RBC	4.1	3.8-5.2 x10^12^/l
Hemoglobin	14	13-18 (g/dL)
HCT	41	35-46%
MCV	87	77-95 fl
MCH	31	26-32 (pg)
MCHC	30	32-36 (g/dL)
Platelets	207	150-400 x10^9^/L
Neutrophils	89	40-80%
Lymphocytes	37	20-40%
Renal Function Tests		
Urea	45	10-50 mg/dl
Serum Creatinine	0.8	0.5-0.9 mg/dl
Liver Function Tests		
Bilirubin total	1.1	0.3-1.2 mg/dl
ALT	37	Up to 40 U/L
AST	40	Up to 40 U/L
ALP	99	40-120 U/L
Serum Electrolytes		
Sodium	141	135-145 mmol/L
Potassium	4.2	3.5-5 mmol/L
Chloride	105	98-107 mmol/L

The raised white blood cell (WBC) count and neutrophilia suggested an ongoing infective process, prompting further evaluation. A computed tomography (CT) scan was performed to delve deeper into the differential diagnoses and discern the exact nature of the infection.

**Figure 1:**
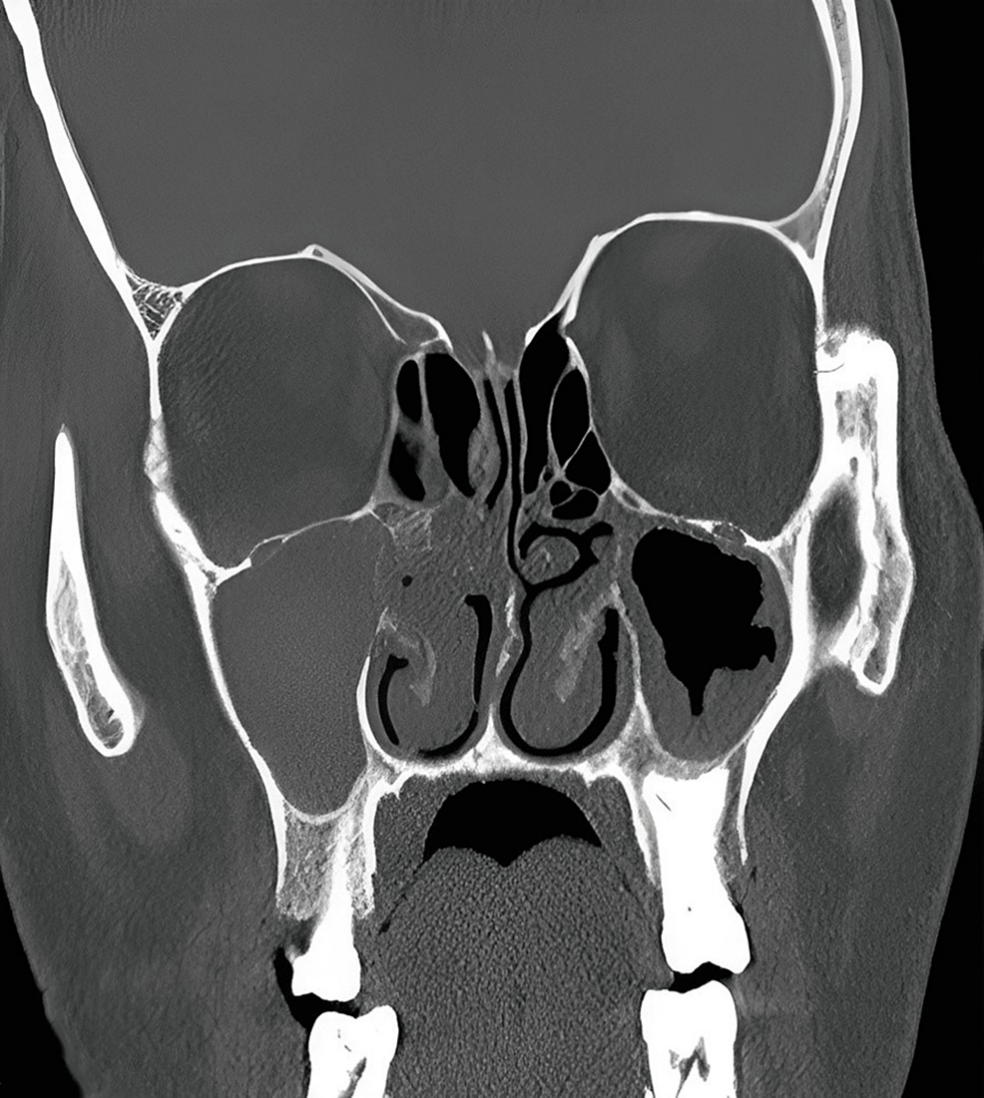
CT scan of the paranasal sinuses. Opacification of the maxillary sinus, ethmoid sinus, middle meatus, and turbinates is evident and more pronounced on the right side. CT: Computed tomography

A CT scan of the brain revealed opacification and thickening in the right ethmoid and maxillary sinuses, hinting at potential fungal or bacterial presence or inflammation-induced thickening. Partial opacification of the right orbit suggested orbital cellulitis, supported by the observed stranding of the orbital fat. Enlarged extraocular muscles in the right orbit further indicated inflammation or direct fungal invasion, aligning with symptoms of a severe fungal infection, likely mucormycosis. CT scan imaging has been provided in Figure 1.

**Figure 2:**
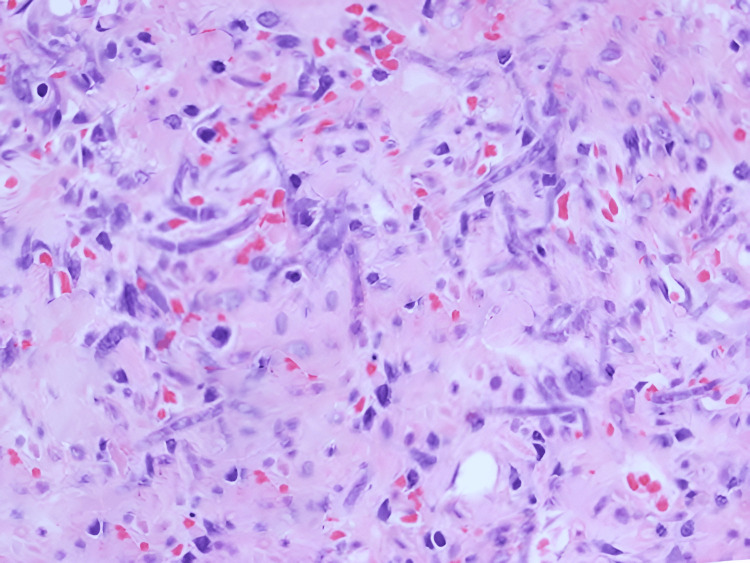
Histopathological examination of patient samples revealed broad, ribbon-like fungal hyphae branching at roughly 90-degree angles, indicative of mucormycosis.

Histopathological examination of patient samples revealed characteristic broad, ribbon-like fungal hyphae branching at roughly 90-degree angles, indicative of mucormycosis. Necrotic areas and fungal angioinvasion with hyphae infiltrating blood vessels strengthened the diagnosis. An evident inflammatory response with neutrophils and macrophages suggested the body's defense against the infection. Gomori Methenamine Silver (GMS) positivity, a stain specific for fungi, confirmed mucormycosis, and cultures showed growth of mucorales mold, with the exact species contingent on the causative pathogen. Histopathological findings are evident in Figure 2.

A comprehensive treatment plan was formulated after a thorough review of the patient's history and extensive evaluations. The primary objective was to manage the patient's underlying diabetes and hypertension while effectively curbing the fungal growth in his eye. Central to this regimen was the initiation of antifungal therapy using Amphotericin B, administered intravenously at a dose of 5-10 mg/kg/day, complemented by surgical debridement to address the localized infection. Concurrently, strict glycemic control was emphasized, with insulin therapy prescribed at 20 units twice daily. For hypertension management, an ACE inhibitor, enalapril (5 milligrams once a day), known for its renal protective properties and efficacy in diabetic patients, was administered.

Supportive care played a vital role in the patient's treatment. This included nutritional support from a dietitian to tailor a diet conducive to immune function and wound healing, considering the patient's diabetic status. Pain management was carefully handled with appropriate analgesics to alleviate discomfort from the condition and post-surgical recovery. Attention was also given to hydration and electrolyte balance, crucial due to the potential renal effects of Amphotericin B, necessitating intravenous fluid administration. Post-surgical wound care involved regular dressing changes and infection monitoring. Ophthalmological support was provided through regular assessments to monitor the condition of the eye and any vision changes. Psychological support, including counseling services, was offered to help the patient cope with the stress and anxiety associated with his condition. Additionally, educational support was provided to the patient and his family regarding disease management, medication adherence, and necessary lifestyle modifications for diabetes and hypertension.

It is important to note the absence of post-surgical images in our case due to limitations in our clinical setting. Throughout the treatment course, these comprehensive supportive care measures ensured all patient needs were addressed. Currently, the patient remains under vigilant supervision, with ongoing management strategies to effectively handle his condition.

## Discussion

Rhinocerebral mucormycosis primarily affects the sinuses and can extend to the brain. Notably, it represents one of the most frequently encountered manifestations of mucormycosis. However, ocular involvement in this condition is rare, with studies suggesting it occurs in a smaller subset of cases. For instance, ocular involvement has been reported in approximately 16%-25% of mucormycosis cases, making its diagnosis and management a clinical challenge [4]. In the context of our patient, the involvement of his right eye, particularly the resulting blurring of vision, underscores the distinctiveness of this case.

Opportunistic fungi primarily drive mucormycosis. These fungi typically exploit hosts with compromised immune systems, making individuals with conditions such as various hematological malignancies, including leukemia, lymphoma, and multiple myeloma, as well as those with organ transplant histories, long-standing uncontrolled diabetes, prolonged steroid use, and acquired immunodeficiency syndrome, particularly susceptible [5]. Impaired immune surveillance in these conditions provides a conducive environment for the aggressive proliferation of the fungus. Reflecting on our patient's history, his longstanding diabetes and hypertension indeed created a favorable milieu for the onset of this fungal infection.

In discussing the unique aspects of this case, it is crucial to understand the typical markers of sinusitis, which are often relied upon for the early diagnosis of sinus-related infections, including mucormycosis. Standard sinusitis markers include symptoms such as nasal congestion or obstruction, purulent nasal discharge, facial pain or pressure, and a reduced sense of smell and taste. Fever and headaches are also common. These symptoms are indicative of inflammation and infection within the sinus cavities. However, in our case, the patient exhibited an atypical presentation, with the absence of these standard sinusitis markers leading to initial diagnostic challenges. The patient's primary symptoms were orbital, including acute vision loss and eye swelling, without the classic sinusitis signs. This atypical presentation is particularly noteworthy in the context of mucormycosis, which can often involve the sinuses and extend to the orbital and cerebral regions. Thus, our case underscores the necessity of maintaining a high index of suspicion for mucormycosis in atypical presentations, especially in patients with risk factors such as diabetes and hypertension.

In managing mucormycosis, particularly in diabetic patients, a comprehensive and multifaceted approach is required. At the forefront of this treatment is antifungal therapy, with Amphotericin B being the primary choice, administered intravenously. The dosing of Amphotericin B can vary based on the severity and location of the infection, as well as the patient’s overall health, including kidney function. Typically, the treatment begins with a lower initial dose, around 0.25 to 0.5 mg/kg per day, to minimize the risk of infusion-related reactions and nephrotoxicity. Based on the patient's response and tolerance, this dose is usually increased gradually to a standard therapeutic dose ranging from 1.0 to 1.5 mg/kg daily. In severe cases, doses may be escalated to up to 1.5 mg/kg per day. However, this higher dosage is associated with an increased risk of side effects, particularly affecting the kidneys. The duration of treatment with Amphotericin B is often extensive, lasting several weeks to months, and is determined by the response to therapy and resolution of the infection. If the initial treatment proves ineffective, posaconazole can be introduced as a salvage therapy. Surgical intervention is often vital, especially in cases presenting with necrotic tissue. Debulking procedures aim to remove necrotic tissue and resect all infected or devitalized areas, facilitating improved penetration of antifungal agents [6].

Equally crucial is strict glycemic control. Patients need close blood glucose monitoring since elevated serum glucose levels can foster fungal growth [7]. In our case, the emphasis on strict glycemic control is particularly pertinent, considering how elevated serum glucose levels can foster fungal growth in infections like mucormycosis. Fungi utilize glucose as a primary nutrient source, and elevated blood glucose levels provide an abundance of this essential nutrient, facilitating rapid fungal proliferation [8]. This aspect is critical to our patient’s management, given his longstanding diabetes.

Furthermore, hyperglycemia is known to impair neutrophil function, a key component of the immune system’s defense against fungal infections. In hyperglycemic conditions, neutrophils may exhibit reduced migration to the site of infection and a diminished capacity to engulf and destroy fungal cells. Additionally, high glucose levels can compromise the phagocytic activity of macrophages, another type of white blood cell crucial in fighting off infections. This impairment of the immune response is a significant concern in managing mucormycosis. Chronic hyperglycemia also leads to changes in microvascular blood flow, potentially reducing the delivery of immune cells and antifungal agents to the site of infection. Moreover, the altered acid-base balance caused by high blood glucose can create an environment more conducive to fungal growth, as some fungi thrive in slightly more acidic conditions.

Therefore, maintaining blood glucose levels within a normal range is not just about managing diabetes but also about limiting the growth and spread of fungi. This approach enhances the effectiveness of antifungal treatments and supports the overall immune response, which is critical in the successful management of mucormycosis, as seen in our patient. The multidisciplinary approach to treatment, including vigilant blood glucose monitoring and adjustments in diabetic therapy, played a vital role in mitigating these risks and contributing to the positive outcome of the case.

Alongside these measures, another potential avenue of treatment we considered is hyperbaric oxygen therapy (HBOT). HBOT involves breathing pure oxygen in a pressurized chamber, significantly increasing oxygen delivery to the tissues. This enhanced oxygenation has several therapeutic benefits, especially pertinent in our case. First, it improves tissue oxygenation in areas with compromised blood flow, which is crucial for areas affected by mucormycosis. Second, the antimicrobial effect of high oxygen levels can directly inhibit fungal growth, as mucormycetes often thrive in low-oxygen environments. Additionally, HBOT aids in wound healing, particularly post-surgical debridement, and reduces edema in infected areas, improving overall treatment efficacy [9].

The protocol for HBOT can vary but typically involves multiple sessions or 'dives' in the hyperbaric chamber, each lasting about 90 to 120 minutes. The total number of sessions might range from 20 to 40 or more, spread over several weeks, depending on the severity of the infection and the patient's health status [10]. This treatment option was explored to enhance our comprehensive management strategy, considering its potential to deter fungal proliferation and support tissue healing.

Blood pressure management, too, plays an essential role, especially for hypertensive individuals. Lastly, the patient must be followed. Regular visual assessments combined with orbital CT or MRI scans can offer insights into treatment progress and the possibility of reversing vision loss.

## Conclusions

Our report underscores a rare instance of orbital mucormycosis in a diabetic and hypertensive patient, highlighting the disease's resilience and deceptive presentation. The absence of standard sinusitis markers delayed early detection, a crucial lapse illuminated by the revealing orbital CT findings. Though isolated orbital mucormycosis is rare, it demands swift diagnostic and therapeutic action. This case emphasizes the need for heightened vigilance in vulnerable populations and a proactive, multidisciplinary approach. In essence, our account not only serves as a clinical alert but also enriches the literature on this elusive fungal foe.
